# JA-mediated H_2_O_2_ and ABA signaling enhances root hydraulic conductance in cotton under partial root-zone irrigation

**DOI:** 10.3389/fpls.2026.1784771

**Published:** 2026-04-02

**Authors:** Zhen Luo, Yong Hui Ma, Chen Yang Li, Wei Tang, He Quan Lu, Xiang Qiang Kong

**Affiliations:** 1 Institute of Industrial Crops, Shandong Academy of Agricultural Sciences, Jinan, China; 2 School of Horticulture, Ludong University, Yantai, Shandong, China

**Keywords:** ABA, H_2_O_2_, JA, partial root-zone irrigation, root hydraulic conductance

## Abstract

**Introduction:**

Partial root-zone irrigation (PRI) is a water-saving technique that improves water use efficiency by inducing physiological adaptations. While abscisic acid (ABA)-mediated stomatal closure from dried roots conserves water (“reducing outflow”), hydraulic compensation in the hydrated roots (“enhancing inflow”) is critical for maintaining plant water balance. The signaling network, particularly the interplay between jasmonic acid (JA), ABA, and hydrogen peroxide (H_2_O_2_), regulating this compensation remains elusive.

**Methods:**

To dissect this signaling network, cotton seedlings were grown in a stratified rhizobox system simulating PRI. Exogenous JA, ABA, H_2_O_2_,and their biosynthesis inhibitor 5,8,11,14‐eicosatetraynoic acid (ETYA), fluridone and diphenyleneiodonium chloride (DPI) were employed to establish causality. Physiological (root water status, hydraulic conductance *L*), JA, ABA and H_2_O_2_ content, and the expression level of their biosynthesis genes and *GhPIP* genes in distinct root zones were analyzed.

**Results:**

Compared to uniformly irrigated or droughted roots, hydrated roots under PRI exhibited significantly enhanced root *L* and upregulated expression of plasma membrane intrinsic protein genes (*GhPIP1;5*, *GhPIP2;8*). This was associated with elevated JA/JA-Ile and H_2_O_2_ levels in the hydrated zone. Exogenous JA and H_2_O_2_ mimicked these effects, while their inhibitors suppressed them. JA was found to be upstream, positively regulating H_2_O_2_ production and modulating local ABA homeostasis. ABA affected root *L* without altering PIP transcript levels. Complementation assays confirmed the specificity of the JA-H_2_O_2_-PIP pathway.

**Conclusion:**

The enhanced hydraulic conductance in the hydrated roots under PRI is an active process orchestrated by a JA-centered signaling network. JA promotes H_2_O_2_ accumulation, which up-regulates *PIP* expression, and fine-tunes ABA levels for potential post-translational regulation. This study elucidates the “enhanced inflow” mechanism in PRI, providing insights for optimizing water-saving irrigation strategies.

## Introduction

Global water scarcity is intensifying due to climate change and increasing population pressures, necessitating the adoption of more efficient irrigation practices. In this context, partial root-zone irrigation (PRI) has been recognized as an effective water-saving strategy that can maintain crop yield while enhancing water use efficiency (Kang et al., 1997; Kang and Zhang, 2004; Fu et al., 2017). Conventional PRI operates by alternately wetting two halves of the root zone, thereby maintaining controlled soil moisture deficits and inducing physiological adaptations that improve drought tolerance (Kang and Zhang, 2004; Iqbal et al., 2020). However, traditional furrow-based PRI systems are often labor-intensive and can lead to non-uniform water distribution, which limits their widespread application. The integration of drip irrigation with PRI has led to an optimized approach termed alternate partial root-zone drip irrigation (APRI). This system combines the precision and controllability of drip irrigation with the water-saving physiological mechanisms of PRI (Topak et al., 2016; Sezen et al., 2019; Hashem et al., 2024; Zhang et al., 2024). A key operational advantage of APRI is its easy implementation, requiring adjustments only to irrigation scheduling and volume. For example, in cotton, an optimized APRI regime has been developed by modifying drip tape layout, setting irrigation intervals at 5–7 days, and alternating between high-volume and low-volume irrigation cycles (Luo et al., 2019a). During high-volume cycles, the entire root zone is irrigated, whereas during low-volume cycles, only the root area adjacent to the drip tape is moistened. This regulated deficit irrigation strategy has been reported to improve irrigation water productivity significantly, with one study in cotton reporting water savings of over 30% without yield compromise (Luo et al., 2019a).

The classic physiological rationale for PRI centers on root-to-shoot signaling. Abscisic acid (ABA) synthesized in the dried root zone is transported to the shoot where it regulates stomatal aperture, thereby improving water use efficiency (Kang and Zhang, 2004; Pérez-Pérez et al., 2018; Slamini et al., 2022). However, the mechanism by which PRI maintains plant water balance involves not only “reducing outflow” (i.e., limiting transpiration) but also critically depends on “enhancing inflow”—specifically, promoting compensatory water uptake by the roots in the hydrated zone. Research indicates that the hydraulic conductance (*L*) of roots in the hydrated zone increases rapidly, which is vital for maintaining normal leaf function and overall plant water status (Wakrim et al., 2005; Li et al., 2007; Wang et al., 2010). This compensatory process is closely associated with up-regulated *PIP* genes and root *L* (McLean et al., 2011). Recent studies have revealed that, in addition to ABA, jasmonic acid (JA) acts as a crucial long-distance signaling molecule, playing a central role in coordinating root–shoot communication and regulating root *L* (Luo et al., 2019b). Under PRI conditions, leaf-derived JA can be transported via the phloem to the roots in the hydrated zone, where it up-regulates the expression of plasma membrane intrinsic protein (PIP) aquaporin genes (e.g., *GhPIP1;5*, *GhPIP2;2*, *GhPIP2;8*), thereby significantly enhancing root *L*. This regulatory network likely involves complex interactions between JA and other signaling molecules, like ABA and hydrogen peroxide (H_2_O_2_). Nevertheless, the precise regulatory network controlling *PIP* gene expression and *L* in the hydrated roots under PRI, particularly the upstream transcriptional regulation, remains to be fully elucidated.

When plants experience water stress, they initiate the production of signaling molecules to modulate root *L* and enhance water uptake. Research indicates that ABA, JA, H_2_O_2_, ethylene and so on are all involved in regulating root *L*. Among these, the role of ABA in modulating water transport pathways has been widely studied, yet its effects are complex: exogenous ABA can increase root *L* (Hose et al., 2000; Sauter et al., 2002; Schraut et al., 2005; Sharipova et al., 2016), or have no significant effect (Aroca et al., 2003). These apparent contradictions may be reconciled by a nonlinear dose–response curve to exogenous ABA (Dodd, 2013). Further evidence indicates that ABA regulates water transport largely by influencing the expression and activity of aquaporins (AQPs). ABA can modulate AQP gene expression at the transcriptional level and also regulate AQP activity via post-translational modifications such as phosphorylation, thereby altering membrane water permeability (Maurel et al., 2008; Gautam and Pandey, 2021). For instance, in maize, root ABA concentration correlates positively with AQP gene expression and root *L* (Parent et al., 2009). Under moderate drought, ABA signaling upregulates *PIP* gene expression to adjust water uptake (Shahzad et al., 2023). More recently, maintaining elevated ABA levels has been shown to be crucial for sustaining AQP abundance, *L*, and growth in barley under drought conditions (Akhtyamova et al., 2024).

In PRI, JA acts as an important long-distance signal that can be translocated via the phloem to roots in the hydrated root zone, where it up-regulates *PIP* gene expression and significantly enhances root *L* (Luo et al., 2019a). Concurrently, H_2_O_2_ serves as a key oxidative signaling molecule in root water perception and transport regulation. It is capable of directly modulating AQP activity and likely engages in crosstalk with JA and ABA pathways (Kong et al., 2016; Sharipova et al., 2021). However, the signaling network that regulates the water-uptake in the hydrated root, particularly the interactions (synergistic or antagonistic) between key signaling molecules like JA, ABA, and H_2_O_2_ in the hydrated root zone, remains poorly defined. Therefore, this study aims to elucidate the molecular and physiological mechanisms by which JA, ABA, and H_2_O_2_ interact to regulate *PIP* expression and root *L* in the hydrated root zone under PRI. The findings are expected to reveal the underlying signaling network that enhances crop water-use efficiency under PRI.

## Materials and methods

### Plant material and growth conditions

Seeds of a commercial upland cotton (*Gossypium hirsutum* L. cv. SCRC37) were subjected to acid delinting using concentrated sulfuric acid (100 mL per kg seeds) for 1–2 min with continuous stirring. Following thorough rinsing six times with tap water, the seeds were sun-dried for 2–3 days. The delinted seeds were sown at a depth of 2 cm in sterilized wet sand contained within plastic boxes (60 cm × 45 cm × 15 cm). The boxes were maintained in a greenhouse under a 16/8 h light/dark photoperiod, with a photosynthetically active radiation intensity of 400 μmol m^-2^ s^-1^ and a temperature of 28 ± 2 °C. Upon full emergence, seedlings were thinned to 100 uniform plants per box. At the two-true-leaf stage, seedlings of uniform size were carefully uprooted, their roots were gently washed with distilled water, and subsequently used for experimental treatments.

### Establishment of the APRI system

#### Experimental design

A stratified rhizobox system was designed to simulate the APRI technique under mulched drip irrigation conditions (**Supplementary Figure 1**). The system comprised two compartments: an hydrated compartment (20 cm × 20 cm × 5.5 cm) and a lower compartment (20 cm × 30 cm × 5.5 cm).

### Irrigation treatments

The experiment comprised three primary soil moisture regimes:

Full Irrigation (FI): Both the upper and lower compartments were maintained at 85% FC with synchronous irrigation every 5 days. This served as the well-watered control.

Alternate Partial Root-zone Irrigation (APRI): For the APRI treatment, soil moisture was managed in an alternating cycle. The upper zone underwent a 5-day moisture cycle between 85% and 55% of field capacity (FC). In contrast, the lower zone followed a 10-day cycle between the same moisture endpoints. The irrigation schedule was as follows: on day 1 (low-volume event), only the upper (hydrated) zone was irrigated to 85% FC; on day 6 (high-volume event), both the upper (hydrated) and lower (dehydrated) zones were uniformly irrigated to 85% FC. The weight of each rhizobox was monitored daily to precisely maintain the target soil moisture levels.

Deficit Irrigation (DI): Both compartments underwent synchronous drying cycles, with irrigation applied only when the soil moisture in both reached 55% FC, thereby maintaining a uniform, sub-optimal water status throughout the root zone. This treatment served as the drought stress control to contrast with the spatially heterogeneous stress of APRI.

The stratified design with differential cycling (5-day for hydrated compartment, 10-day for dehydrated compartment) aimed to simulate field conditions under mulch drip irrigation. In order to control the moisture, soil samples (approximately 30 g each) were collected from each treatment plot using a standard soil core sampler. These samples were then oven-dried at 105°Cuntil a constant mass was achieved to determine their water content. Based on this measured initial moisture, the corresponding 30-g soil samples were re-wetted to restore their original field water content. They were subsequently returned to their exact sampling locations to preserve the site-specific soil structure. Following this, the entire soil volume within the experimental plots was adjusted to the target moisture level by compensating for the calculated water deficit. A fixed irrigation interval of 5 days was implemented to maintain the desired soil water regime, a schedule that was determined through preliminary experiments.

### Chemical treatments

Based on prior evidence suggesting that JA may act as a leaf-derived signal in PRI (Luo et al., 2019b), JA and its biosynthesis inhibitor 5,8,11,14‐eicosatetraynoic acid (ETYA) were applied via foliar spraying. In contrast, ABA, H_2_O_2_, and their respective inhibitors (fluridone and diphenyleneiodonium chloride, DPI) were supplied via the nutrient solution to the roots in the hydrated compartment. All the chemicals except H_2_O_2_ were first dissolved in absolute ethanol to prepare 10 mM stock solutions. JA and ETYA were diluted in distilled water to achieve the following final concentrations: 100 µM JA and 100 µM ETYA, and then sprayed on the leaves. The same volume distilled water containing an equivalent volume of ethanol (0.5%, v/v) served as the vehicle control. Other stocks were then diluted in nutrient solution to achieve the following final concentrations: 200 µM ABA, 10 µM fluridone, and 200 µM DPI. A nutrient solution containing an equivalent volume of ethanol (0.5%, v/v) served as the vehicle control. A nutrient solution containing 500 µM H_2_O_2_ was supplied to the roots in the hydrated compartment directly.

### Plant sampling

Following 24 h of chemical treatment (accompanied by low-volume event), root tissues from both the hydrated and lower compartments, along with leaves, were collected from six seedlings per treatment. Samples were rinsed three times with distilled water, gently blotted dry with filter paper, immediately frozen in liquid nitrogen, and stored at -80 °C until further analysis.

### Physiological measurements

#### Root relative water content

Root samples (0.5-1.0 g) collected between 9:00 and 10:00 AM were used. After surface-drying, fresh weight (FW) was recorded. Roots were then immersed in distilled water at 4 °C overnight to attain turgid weight (TW). The dry weight (DW) was determined by lyophilization (freeze-drying). Briefly, the turgid roots were rapidly frozen in liquid nitrogen and then transferred to a freeze-dryer. The samples were pre-frozen at -80 °C for at least 2 hours to ensure complete solidification. Primary drying was conducted under a vacuum (approximately 10–30 Pa) with the condenser temperature below -50 °C for 48 hours, followed by a secondary drying phase if necessary, until a constant weight was achieved. The use of lyophilization, instead of conventional oven-drying, was to prevent potential thermal degradation of cell wall components (e.g., cellulose) at high temperatures, thereby ensuring the accuracy of DW measurement and subsequent calculations of water content parameters (Stegen et al., 1998). RRWC was calculated as: RRWC (%) = [(FW - DW)/(TW - DW)] × 100.

#### Root water potential and hydraulic conductance (*L*)

Root water potential and *L* were measured using a pressure chamber (PMS Model 670) as described by Sánchez-Romera et al. (2014). Measurements were conducted between 11:00 AM and 3:00 PM. The root system from each compartment was excised separately, sealed into the chamber lid with the cut stump protruding, and submerged in nutrient solution. The balancing pressure required to exude sap from the cut surface was recorded as root water potential. For *L* measurement, a graduated micropipette was attached to the stump to collect xylem sap exudate. After applying pressure equal to the root water potential, a series of incremental pressures were applied. The sap flow rate (mg g^-1^ DW h^-1^) was plotted against the applied pressure (MPa). The slope of the linear regression provided the root *L* value (mg g^-1^ DW h^-1^ MPa^-1^). Six biological replicates were measured per treatment.

### Extraction and quantification of JA, JA-Ile, and ABA

Hormone extraction and purification were performed based on the method of Fu et al. (2012) with modifications. Approximately 200 mg of frozen tissue was homogenized and extracted in methanol containing deuterated internal standards ([²H_5_]JA, [²H_3_]JA-Ile, [²H_6_]ABA) for 24 h at 4 °C. The extract was centrifuged, and the supernatant was purified using an Oasis MAX solid-phase extraction cartridge. Hormone quantification was performed using an ultra-performance liquid chromatography system (Waters) coupled to a 5500 Q-Trap mass spectrometer (AB Sciex). Separation was achieved on a BEH C18 column with a mobile phase of 0.05% acetic acid in water (A) and 0.05% acetic acid in acetonitrile (B). Analytes were detected in multiple reaction monitoring (MRM) mode. Four biological replicates, each with three technical repeats, were analyzed.

### Determination of H_2_O_2_ content

The H_2_O_2_ concentration in root tissues was determined using an Amplex Red Hydrogen Peroxide/Peroxidase Assay Kit. Frozen root tissue (50 mg) was homogenized in 500 µL of phosphate buffer (20 mM K_2_HPO_4_, pH 6.5). After centrifugation, 50 µL of the supernatant was incubated with 0.2 U mL^-1^ horseradish peroxidase and 100 µM Amplex Red reagent in the dark for 30 min at room temperature. Fluorescence was measured using a microplate reader (FLUOStar Optima) at an excitation/emission of 560/590 nm (Xing et al., 2008).

### Quantitative real-time PCR analysis

Total RNA was extracted from root tissues using TRIzol reagent. First-strand cDNA was synthesized using SuperScript II Reverse Transcriptase. Gene-specific primers were designed with Primer Premier 5.0 software (sequences listed in **Supplementary Table 1**). qRT-PCR was performed on a Bio-Rad IQ2 system using SYBR Green PCR Master Mix. The thermal cycling protocol consisted of an initial denaturation at 95 °C for 3 min, followed by 40 cycles of 95 °C for 10 s, 60 °C for 10 s, and 72 °C for 10 s. Gene expression levels were analyzed using the comparative 2−ΔΔCT method. The threshold cycle (Ct) values were first normalized to the geometric mean of the β-actin gene to obtain ΔCT values. The stability of the β-actin reference gene under the experimental conditions was verified by calculating the coefficient of variation (CV) of its raw Ct values across all samples (**Supplementary Table 1**). The CV was 3.17%, which is below the 5% threshold for stable expression, consistent with previous studies (Li et al., 2023; Clark et al., 2025). The ΔΔCT was then calculated as the difference between the ΔCT of each sample and the mean ΔCT of the full irrigation (FI) control group. The relative expression level (fold change) was finally expressed as 2−ΔΔCT (Livak and Schmittgen, 2001).

### Statistical analysis

The experiment followed a completely randomized design. Each stratified rhizobox containing one plant was considered an independent experimental unit (biological replicate, n=6). All data presented are from a single 24-hour endpoint. Normality was verified using the Shapiro-Wilk test. Homogeneity of variances was assessed using Levene’s test. For parameters where Levene’s test indicated heterogeneity of variances (P < 0.05), we performed Welch’s ANOVA, which is robust to unequal variances. Following a significant main effect (P < 0.05), the Games–Howell *post-hoc* test (which does not assume equal variances) was applied for multiple comparisons. For parameters with homogeneous variances (P ≥ 0.05), one-way ANOVA was used, and *post-hoc* comparisons were also conducted using the Games–Howell test to maintain consistency and robustness across all analyses. The significance level was maintained at α = 0.05 for all tests.

## Results

### PRI maintains favorable root water status and spatially regulates aquaporin expression

**Figure 1:**
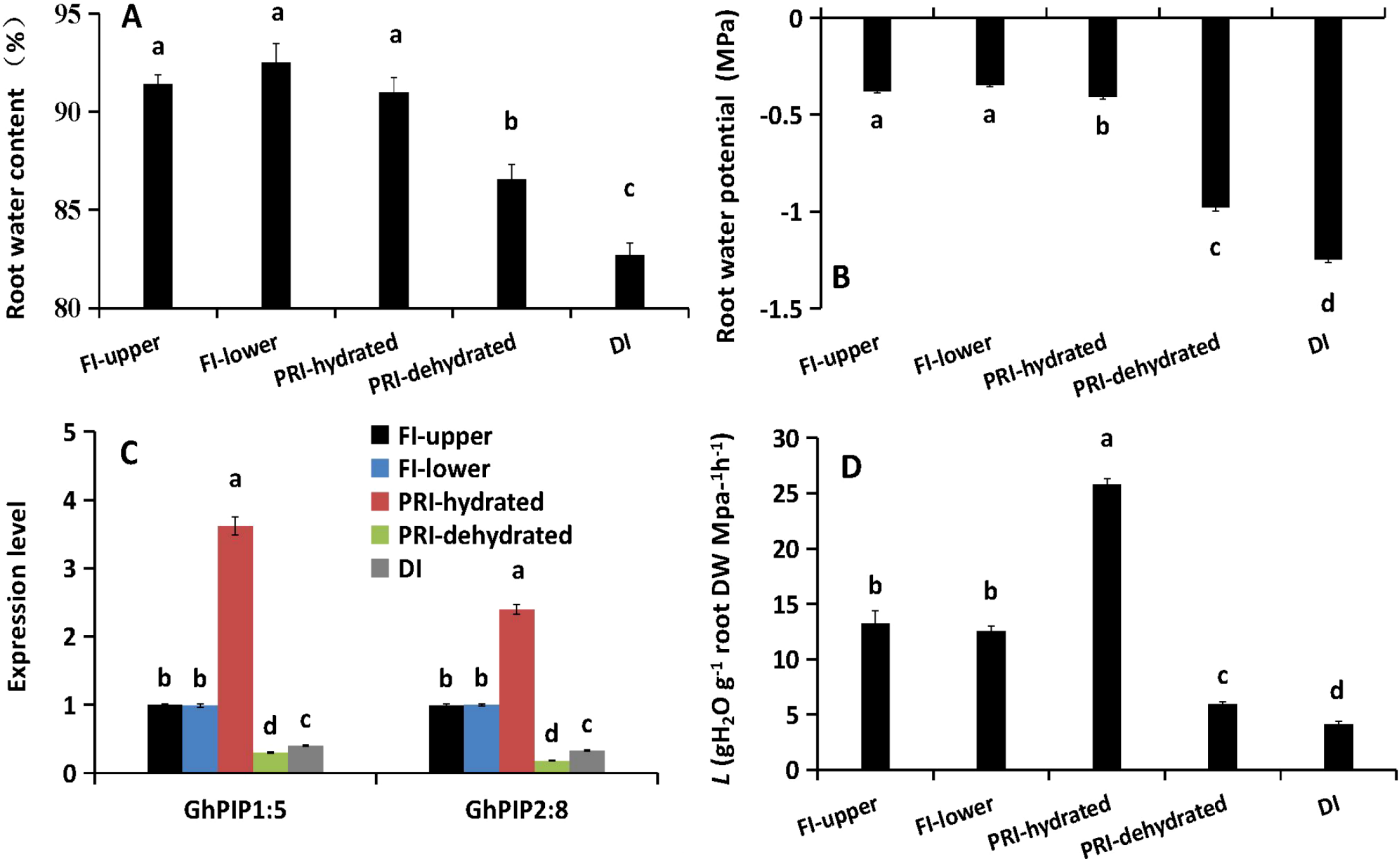
Physiological and molecular responses of cotton roots to different irrigation regimes at 24 HAT. **(A)** RRWC, Root relative water content **(B)** Root water potential (Ψroot). **(C)** Relative expression levels of aquaporin genes GhPIP1;5 and GhPIP2;8. **(D)** Root hydraulic conductance (L). FI, Full Irrigation; PRI, Partial Root-zone Irrigation (hydrated zone, dehydrated zone); DI: Deficit Irrigation. Different lowercase letters indicate statistically significant differences (P < 0.05) among treatments within each parameter as determined by Games-Howell *post-hoc* test. Data are presented as mean ± SD (n = 6 independent rhizoboxes).

Under drought stress, root water content and water potential were significantly reduced in the roots of the DI treatment and the lower (dehydrated) part of the PRI system, compared to the hydrated control and the upper (hydrated) part of PRI (**Figures 1A, B**). Notably, despite experiencing the same soil drought, the dehydrated part of PRI maintained significantly higher water content and water potential (by 4.6% and 21.5%, respectively) than the DI roots. This indicates that the PRI system itself, through likely hydraulic redistribution, conferred a protective advantage to its drought-exposed sectors. Consistent with the water status, the expression levels of aquaporin genes *GhPIP1;5* and *GhPIP2;8* were up-regulated by 3.2- and 2.6-fold, respectively, in the hydrated part of PRI compared to the control. In contrast, their expression was down-regulated more than 2-fold in both the DI roots and the dehydrated part of PRI (**Figure 1C**). Accordingly, root *L* increased by approximately 100% in the hydrated part of PRI but decreased by 53.8% and 67.7% in the dehydrated part of PRI and DI roots, respectively (**Figure 1D**). Together, these results demonstrate that PRI spatially modulates root water status, aquaporin expression, and hydraulic conductance, with the hydrated region exhibiting enhanced water transport capacity while the dehydrated region retains better hydration than DI roots.

### Differential and coordinated accumulation of JA, ABA, and H_2_O_2_ in PRI roots

**Figure 2:**
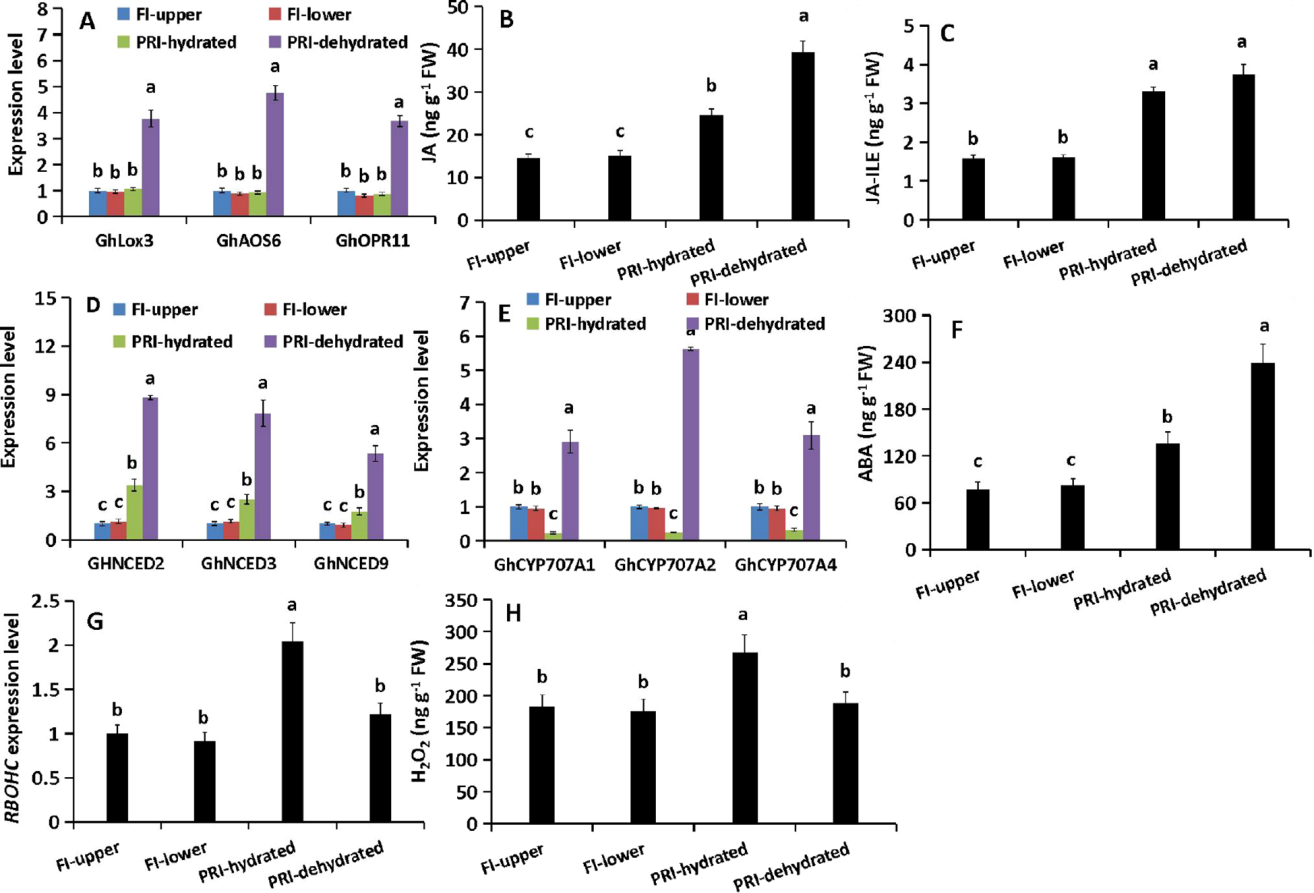
Dynamics of JA, ABA and H_2_O_2_ signaling in cotton roots under different irrigation regimes at 24 HAT. **(A)** Relative expression of JA biosynthesis genes (GhLOX3, GhAOS6, GhOPR11). **(B, C)** Endogenous levels of JA and its bioactive conjugate JA-Ile. **(D)** Relative expression of ABA biosynthesis genes (GhNCED2, GhNCED5, GhNCED9). **(E)** Relative expression of ABA catabolic genes (GhCYP707A1, GhCYP707A2, GhCYP707A4). **(F)** Endogenous ABA content. **(G)** Relative expression of the respiratory burst oxidase homolog gene GhRBOHC. **(H)** Endogenous H_2_O_2_ content. FI, Full Irrigation; PRI, Partial Root-zone Irrigation (hydrated zone, dehydrated zone). Different lowercase letters indicate statistically significant differences (P < 0.05) among treatments within each panel as determined by Games-Howell *post-hoc* test. Data are presented as mean ± SD (n = 6 independent rhizoboxes).

In response to drought at 24 hours after treatment (HAT), the dehydrated part of PRI roots showed a coordinated induction of JA and ABA pathways. The expression of JA biosynthesis genes (*GhLOX3*, *GhAOS6*, *GhOPR11*) increased by 3.77-, 4.76-, and 3.68-fold, respectively, concomitant with 2.67- and 2.34-fold elevations in JA and JA-Ile levels (**Figures 2A–C**). The hydrated part also accumulated more JA and JA-Ile (1.67- and 2.08-fold), albeit without significant changes in the tested biosynthesis genes. ABA biosynthesis genes (*GhNCED2*/*5*/*9*) were strongly induced in the dehydrated part (5.33- to 8.81-fold), and moderately in the hydrated part (1.75- to 3.39-fold) (**Figure 2D**). Conversely, the expression of ABA catabolic genes (*GhCYP707A1*/*2*/*4*) increased in the dehydrated part (2.91- to 5.63-fold) but decreased by 3.13- to 4.34-fold in the hydrated part (**Figure 2E**). This distinct regulation of biosynthesis and catabolism resulted in a 3.01-fold increase in ABA content in the dehydrated part and a 1.70-fold increase in the hydrated part (**Figure 2F**). H_2_O_2_ levels and its biosynthetic gene *RBOHC* were specifically elevated (1.48- and 2.14-fold) only in the hydrated part of PRI (**Figures 2G, H**). These data reveal a spatially partitioned hormonal and oxidative response in PRI roots, with the dehydrated zone intensively activating both JA and ABA synthesis, while the hydrated zone uniquely accumulates H_2_O_2_ alongside milder hormone increases.

### JA and H_2_O_2_ positively regulate aquaporin expression and root *L*

**Figure 3:**
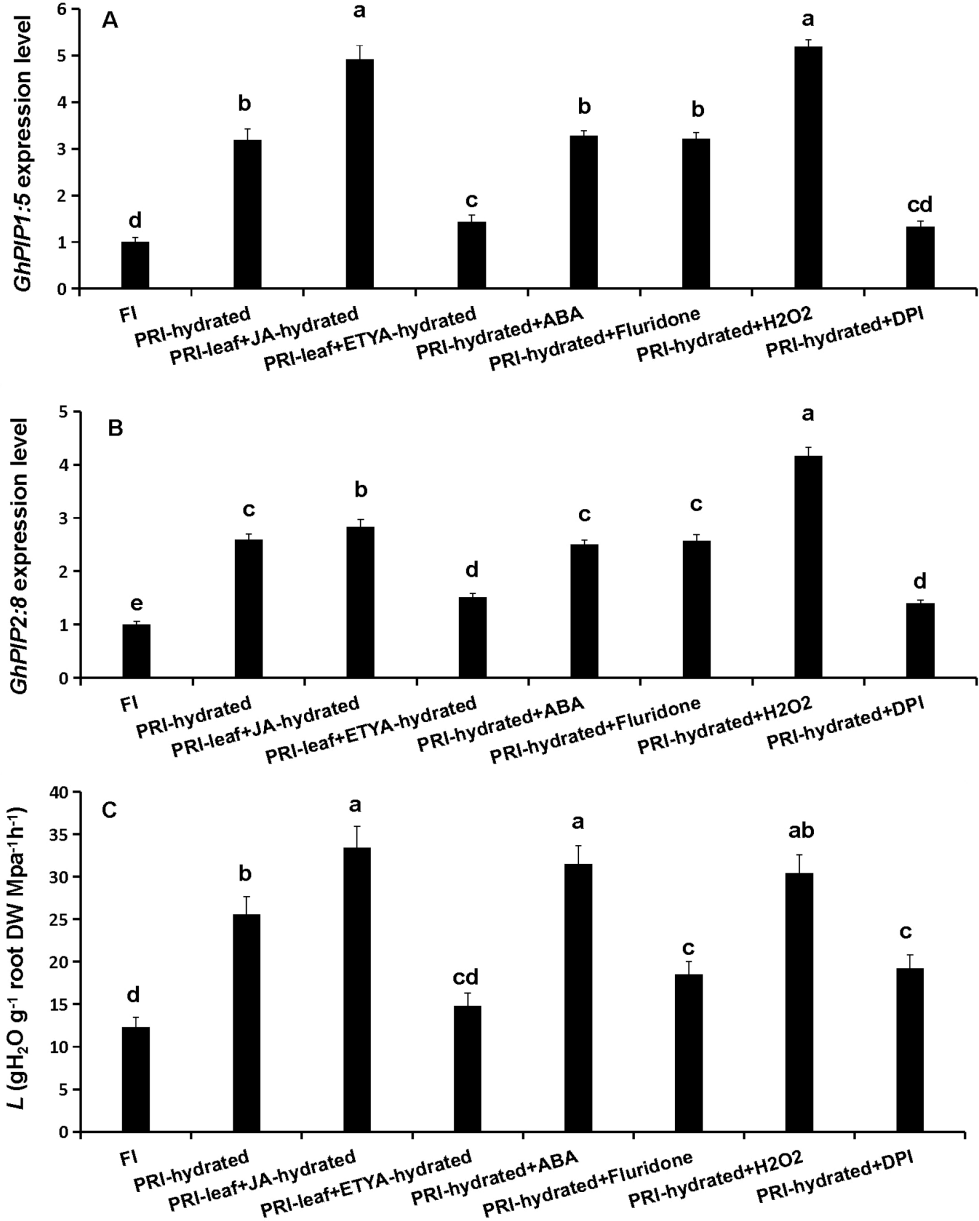
Effects of pharmacological treatments on aquaporin expression and hydraulic conductance in the hydrated root zone under PRI. Roots in the hydrated compartment were treated with exogenous compounds or inhibitors via root application (ABA, Fluridone, H_2_O_2_, DPI) or foliar spray (JA, ETYA). **(A, B)** Relative expression levels of GhPIP1;5 and GhPIP2;8. **(C)** Root hydraulic conductance (L). FI, Full Irrigation; PRI, Partial Root-zone Irrigation (hydrated zone, dehydrated zone). Different lowercase letters indicate statistically significant differences (P < 0.05) among treatments within each panel as determined by Games-Howell *post-hoc* test. Data are presented as mean ± SD (n = 6 independent rhizoboxes).

To test causality, exogenous JA, ABA and H_2_O_2_, and their respective inhibitors were applied to the hydrated roots of PRI, with the exception that the JA antagonist ETYA was sprayed on the leaves. Exogenous JA and H_2_O_2_ significantly up-regulated *GhPIP1;5* and *GhPIP2;8* expression (by 2.83- to 5.19-fold) and increased root *L* (by 23.8-36.2%). Their respective inhibitors (ETYA and DPI) produced opposite effects, significantly down-regulated *GhPIP1;5* and *GhPIP2;8* expression (by 1.73- to 2.39-fold) and decreased root *L* (by 42.1 and 24.9%, respectively) (**Figures 3A–C**). This establishes a direct positive regulatory link from JA and H_2_O_2_ to aquaporin-mediated root water transport. While exogenous ABA and its inhibitor (Fluridone) did not alter *GhPIP* expression, they increased and decreased root *L* by 28.2% and 24.6%, respectively (**Figures 3B, C**), indicating that ABA modulates root hydraulics through aquaporin-independent pathways. Thus, JA and H_2_O_2_ are identified as key positive regulators of aquaporin expression and hydraulic conductance in PRI roots under PRI.

### A hierarchical signaling network: JA positively regulates H_2_O_2_ production and ABA homeostasis

**Figure 4:**
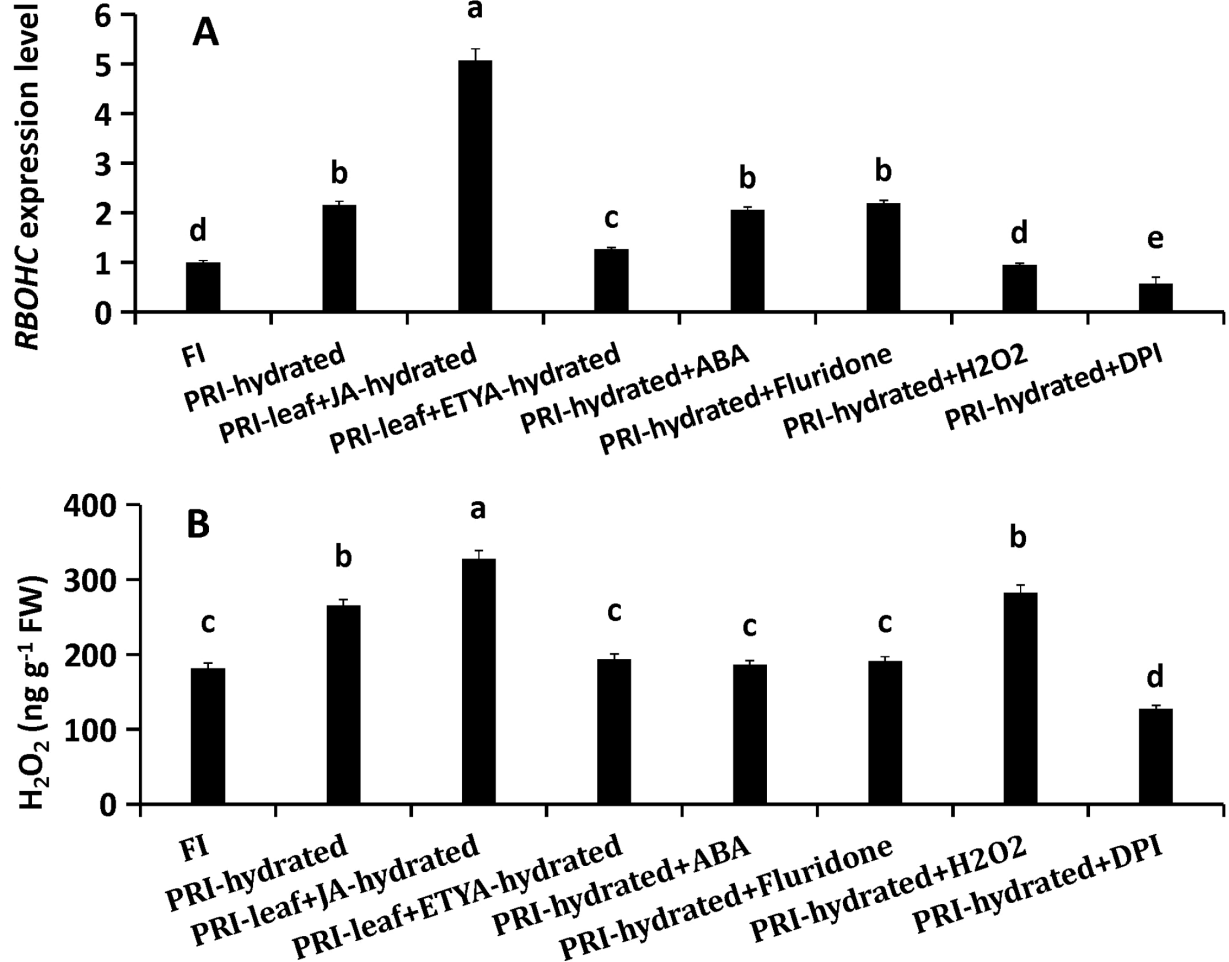
Regulation of H_2_O_2_ production by JA and ABA in the hydrated root zone. Roots in the hydrated compartment were treated with exogenous compounds or inhibitors via root application (ABA, Fluridone, H_2_O_2_, DPI) or foliar spray (JA, ETYA). **(A)** Relative expression of GhRBOHC. **(B)** Endogenous H_2_O_2_ content. FI, Full Irrigation; PRI, Partial Root-zone Irrigation (hydrated zone, dehydrated zone). Different lowercase letters indicate statistically significant differences (P < 0.05) among treatments within each panel as determined by Games-Howell *post-hoc* test. Data are presented as mean ± SD (n = 6 independent rhizoboxes).

**Figure 5:**
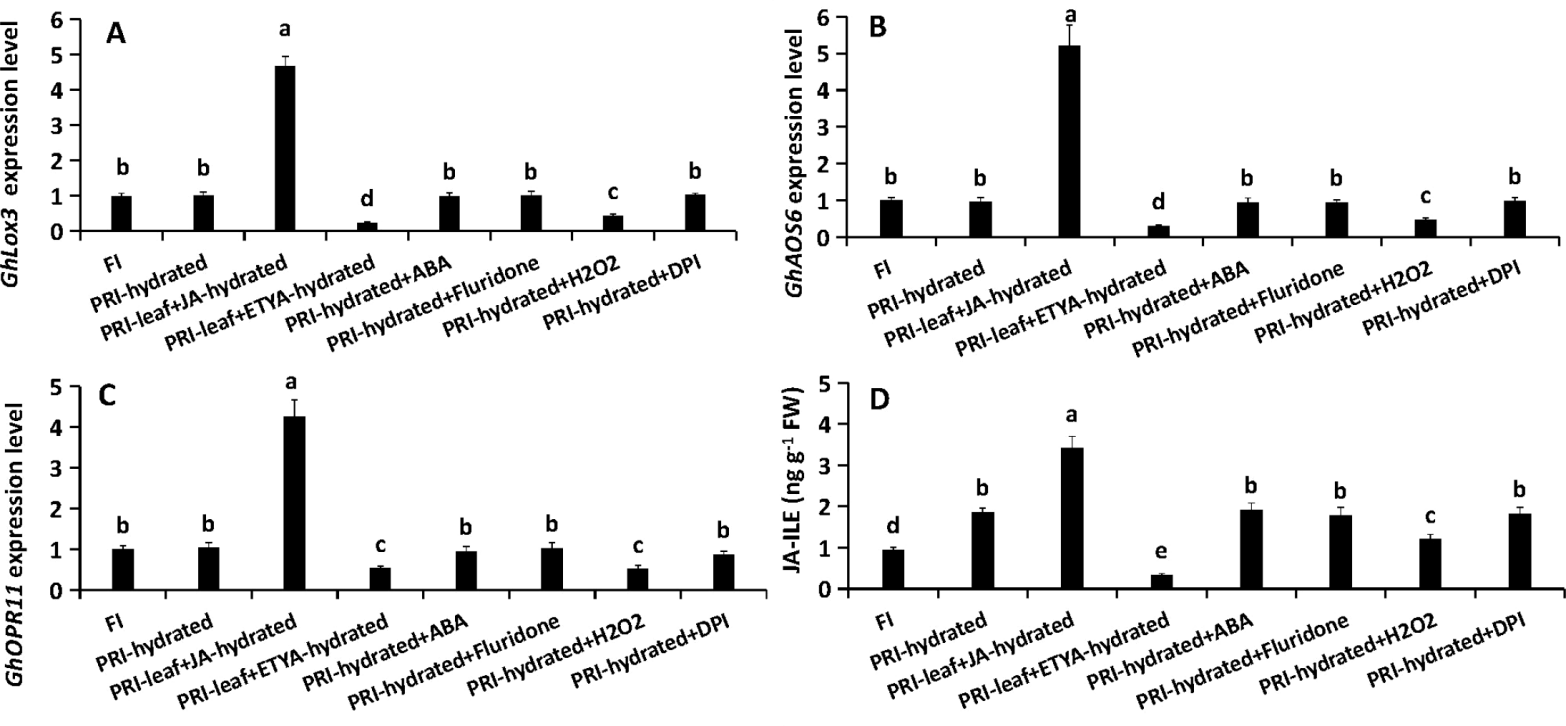
Feedback regulation of JA biosynthesis by H_2_O_2_ in the hydrated root zone. Roots in the hydrated compartment were treated with exogenous compounds or inhibitors via root application (ABA, Fluridone, H_2_O_2_, DPI) or foliar spray (JA, ETYA). **(A-C)** Relative expression of JA biosynthesis genes GhLOX3, GhAOS6, and GhOPR11. **(D)** Endogenous JA-Ile content. FI: Full Irrigation; PRI: Partial Root-zone Irrigation (hydrated zone, dehydrated zone). Different lowercase letters indicate statistically significant differences (P < 0.05) among treatments within each panel as determined by Games-Howell *post-hoc* test. Data are presented as mean ± SD (n = 6 independent rhizoboxes).

**Figure 6:**
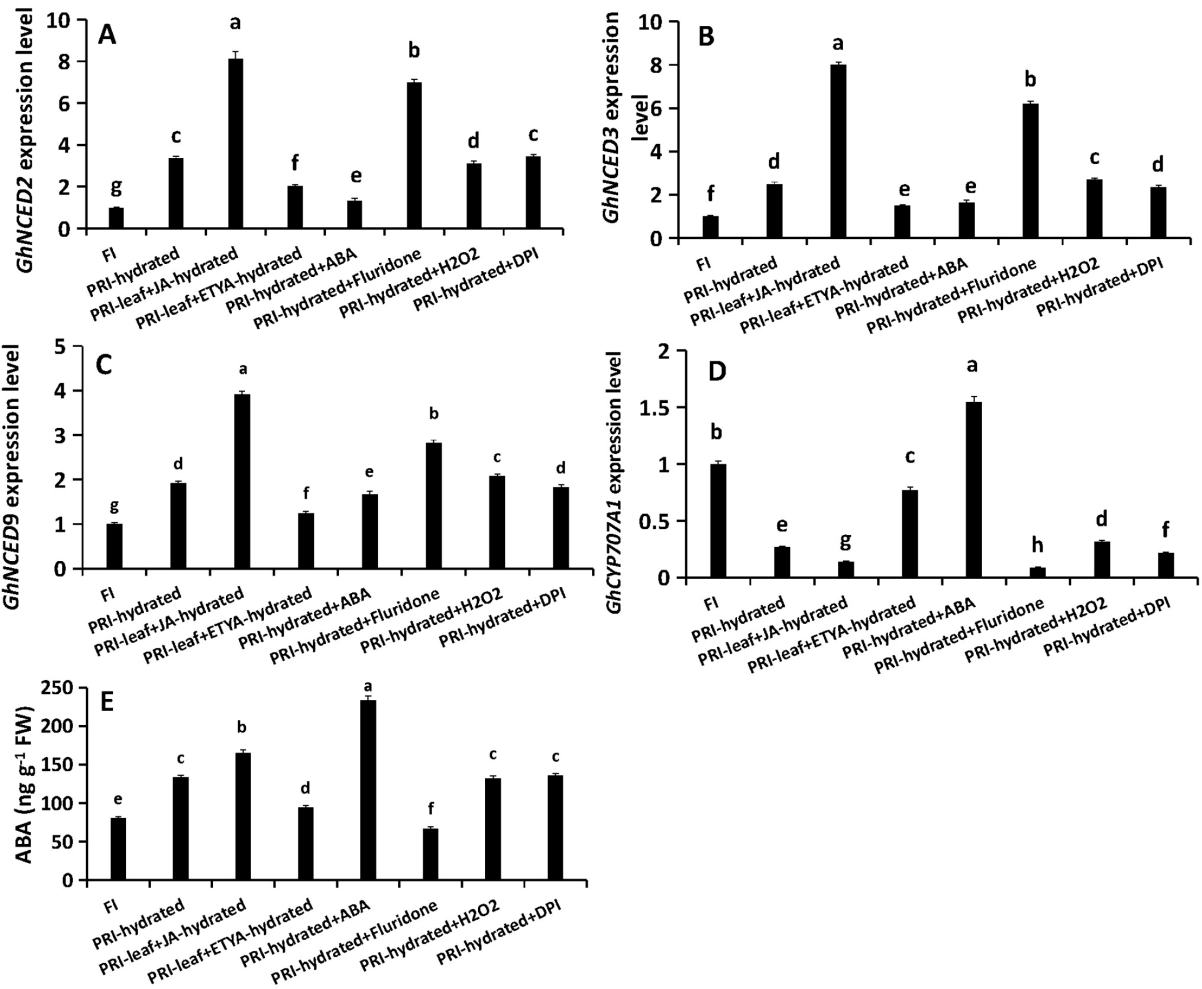
Regulation of ABA homeostasis by JA and H_2_O_2_ in the hydrated root zone. Roots in the hydrated compartment were treated with exogenous compounds or inhibitors via root application (ABA, Fluridone, H_2_O_2_, DPI) or foliar spray (JA, ETYA). **(A-C)** Relative expression of ABA biosynthesis genes *GhNCED2*, *GhNCED3*, and *GhNCED9*. **(D)** Relative expression of the ABA catabolic gene *GhCYP707A1*. **(E)** Endogenous ABA content. FI, Full Irrigation; PRI, Partial Root-zone Irrigation (hydrated zone, dehydrated zone). Different lowercase letters indicate statistically significant differences (P < 0.05) among treatments within each panel as determined by Games-Howell *post-hoc* test. Data are presented as mean ± SD (n = 6 independent rhizoboxes).

The treatments revealed a unidirectional regulation: JA positively regulated H_2_O_2_ production. Exogenous JA increased *RBOHC* expression (by 134.74%) and H_2_O_2_ content (by 23.42%), while ETYA decreased *RBOHC* expression (by 41.1%) and H_2_O_2_ content (by 37.1%) (**Figures 4A, B**). ABA manipulations had no effect on H_2_O_2_. Conversely, H_2_O_2_ negatively fed back on JA synthesis, as its application suppressed JA biosynthetic genes and JA-Ile levels (**Figures 5A–D**). Furthermore, JA signaling positively regulated ABA homeostasis. JA application enhanced the expression of ABA biosynthesis genes *GhNCED2/3/9* by 2.41-, 3.2- and 2.04-fold, respectively, suppressed *GhCYP707A1* by 50%, and elevated ABA content by 23.7%. ETYA had the opposite effects (**Figures 6A–E**). ABA itself triggered a negative feedback loop, down-regulating its own biosynthetic genes and up-regulating *GhCYP707A1*, even as the exogenous hormone elevated total ABA content (**Figures 6A–E**). H_2_O_2_ manipulations did not affect the ABA pathway. These interactions delineate a hierarchical signaling network where JA acts as an upstream activator, promoting both H_2_O_2_ production and ABA accumulation, while receiving negative feedback from H_2_O_2_ and allowing ABA self-regulation.

### Complementation assays validate the specific roles of JA and H_2_O_2_

**Figure 7:**
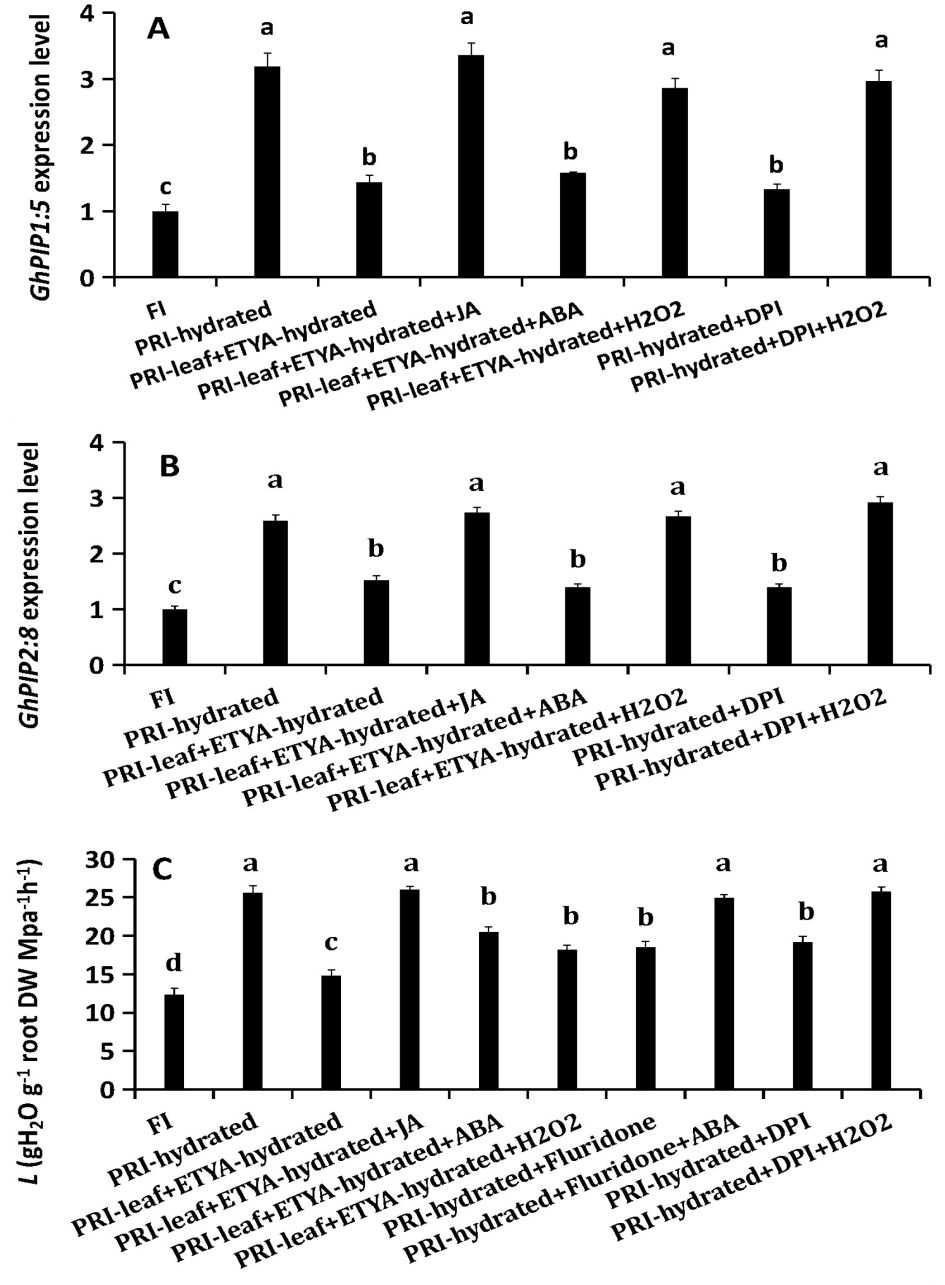
Complementation tests to validate the specificity of the JA-H_2_O_2_ signaling pathway. The hydrated roots were treated with inhibitors (ETYA, DPI, or Fluridone) alone or in combination with their corresponding exogenous signaling molecules (JA, H_2_O_2_, or ABA). **(A, B)** Relative expression of *GhPIP1;5* and *GhPIP2;8*. **(C)** Root hydraulic conductance (*L*).FI: Full Irrigation; PRI: Partial Root-zone Irrigation (hydrated zone, dehydrated zone). Different lowercase letters indicate statistically significant differences (P < 0.05) among treatments within each panel as determined by Games-Howell *post-hoc* test. Data are presented as mean ± SD (n = 6 independent rhizoboxes).

A complementation assay confirmed the specificity of the interactions. The suppressed *GhPIP* expression caused by ETYA was rescued by exogenous JA or H_2_O_2_, but not ABA (**Figures 7A, B**). The reduced root *L* caused by ETYA was rescued by exogenous JA, but partly rescued by exogenous ABA or H_2_O_2_. Similarly, the suppression of *GhPIP* expression and the decrease in root *L* caused by DPI were specifically reversed by the addition of H_2_O_2_. The reduced root *L* caused by Fluridone (ABA inhibitor) was restored by exogenous ABA. These complementation results validate the specific and central roles of JA and H_2_O_2_ in regulating aquaporins, while affirming that ABA operates in a parallel pathway to influence root hydraulics.

## Discussion

The present study elucidates the intricate signaling network orchestrating the enhancement of root *L* in the hydrated compartment of cotton roots under an APRI regime. Our results demonstrate that the improved water status and hydraulic capacity of the hydrated roots under PRI are not a passive consequence of water availability but an active physiological adaptation regulated by a coordinated interplay between JA, H_2_O_2_, and ABA. These findings advance our mechanistic understanding of the “enhanced inflow” component in PRI systems and reveal a hierarchical signaling cascade centered on JA.

### Hydraulic compensation in hydrated roots is an active process driven by JA-mediated AQP regulation

Consistent with our previous findings using a split-root system (Luo et al., 2019b), the stratified rhizobox system employed here confirmed that hydraulic compensation in the irrigated zone of APRI is an active process (**Supplementary Figure 1**). The significant increase in *L* (~100%) and *PIP* expression in hydrated roots, contrasted with their decline in the dried zone and under DI (**Figure 1**), underscores this adaptation. Notably, the elevated JA/JA-Ile levels in hydrated roots without concurrent upregulation of local biosynthesis genes (**Figures 2A–C**) support the hypothesis of leaf-derived JA as a long-distance signal, which was further verified by foliar application of JA and its inhibitor ETYA (**Figure 3**, **Supplementary Figure 2**).

The stark contrast between the two root halves under APRI is instructive. While roots in the drying zone and those under uniform deficit irrigation (DI) suffered declines in water status and *L*, the hydrated APRI roots displayed a remarkable ~100% increase in *L* (**Figure 1**). This demonstrates that hydrated roots actively enhance their *L*—a hydraulic compensation critical for maintaining overall plant water balance. This active “foraging” response in the wet zone is a key physiological pillar supporting the water-use efficiency benefits of PRI (Wakrim et al., 2005; Wang et al., 2010; Hashem et al., 2024), alongside the well-known “water-saving” effect from partial stomatal closure induced by dry-root-sourced ABA.

### JA orchestrates the signaling network: positively regulating H_2_O_2_ and modulating ABA homeostasis

**Figure 8:**
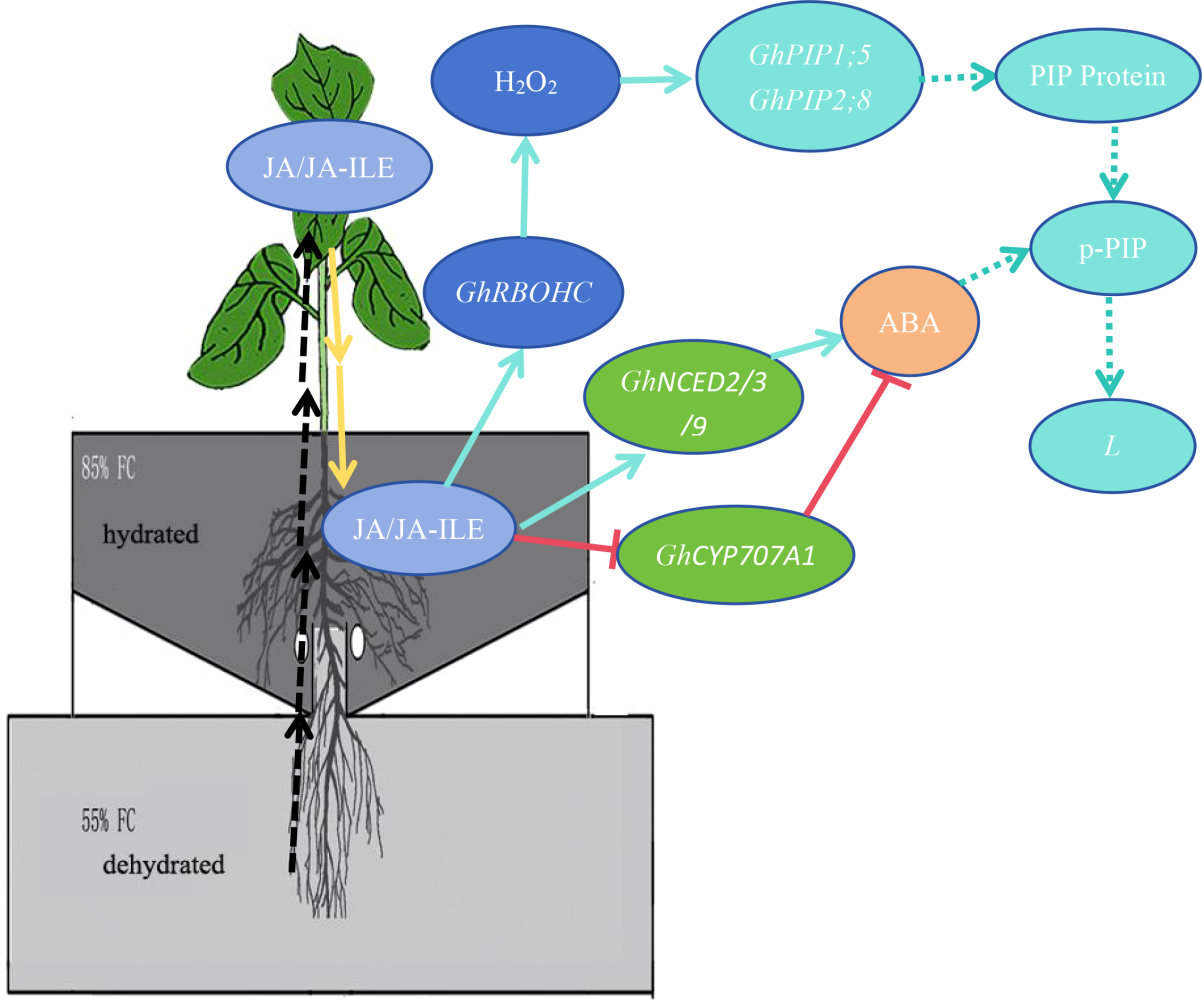
A proposed model for JA-centered signaling network enhancing root hydraulic conductance (L) in the hydrated zone of cotton under PRI, partial root-zone irrigation. Under PRI conditions, JA synthesized in leaves (likely induced by signals from the dried root zone) is translocated to the hydrated roots. In the hydrated zone, JA (1) activates *RBOHC*-mediated H_2_O_2_ production, which up-regulates the expression of plasma membrane intrinsic protein genes (GhPIPs), thereby increasing water influx; and (2) modulates local ABA homeostasis. The accumulated ABA may fine-tune *L* through post-translational regulation of aquaporin activity. Solid arrows indicate pathways supported by direct experimental evidence in this study; dashed arrows indicate hypothesized or indirect relationships.

JA serves as a central regulator orchestrating plant adaptation to diverse abiotic and biotic stresses through complex physiological and molecular adjustments (Wang et al., 2020). Its role in mitigating damage under conditions such as drought has been documented across species, including M. villosum and strawberry (Vieira, 2023; Zhong et al., 2025). In the present study JA played a key role in regulating the water uptake in the hydrated roots of cotton under PRI. The results demonstrated that exogenous JA application directly upregulates the expression of *GhRBOHC*—a gene encoding a critical NADPH oxidase responsible for apoplastic ROS generation—and concomitantly elevates H_2_O_2_ levels. Conversely, inhibition of JA biosynthesis produced the opposite effect (**Figure 4**). This JA-mediated H_2_O_2_ burst is functionally significant, as the application of H_2_O_2_ could partially rescue the suppression of *L* induced by the JA inhibitor ETYA (**Figure 7**). Exogenous JA and H_2_O_2_ significantly up-regulated *GhPIP1;5* and *GhPIP2;8* expression and increased root *L*. Their respective inhibitors (ETYA and DPI) produced opposite effects (**Figures 3A–C**). These findings collectively articulate a sequential pathway: JA signal → *RBOHC* activation/H_2_O_2_ production →up-regulated expression level of *GhPIP* genes →enhancement of root *L* (**Figure 8**). Exogenous H_2_O_2_ enhanced the expression of *GhPIP* genes in the roots in the non-saline side under Non-uniform root salinity (Kong et al., 2016). PIP was identified as an H_2_O_2_ transporter to deal with biotic or abiotic stress (Bienert and Chaumont, 2014; Rhee et al., 2017; Li et al., 2024). This suggests that H_2_O_2_ and PIP may form a self-regulation or signal amplification loop in the regulation of root L.

Simultaneously, JA signaling was found to positively regulate abscisic acid (ABA) homeostasis within the same hydrated root compartment. JA application upregulated key ABA biosynthetic genes (*GhNCEDs)* and repressed a major catabolic gene (*GhCYP707A1*), resulting in increased ABA accumulation (**Figure 6**). This positive interaction between JA and ABA is a recognized phenomenon in stress signaling, where they often act synergistically to amplify adaptive responses. The discovery of transcription factors like PtoMYB99, which concurrently suppresses the biosynthesis of both ABA and JA to diminish osmotic stress tolerance, further underscores the tight coupling of these hormonal pathways (Long et al., 2024).

However, the role of this accumulated ABA in the hydrated zone is distinct and appears to be primarily post-transcriptional. ABA manipulations did not alter *GhPIP* gene expression (**Figure 3B**) but did influence root *L* (**Figure 3C**), and exogenous ABA could partially rescue the root *L* reduction caused by JA inhibition (**Figure 7C**). This strongly suggests that ABA regulates root *L* through mechanisms independent of *PIP* transcript abundance, such as post-translational modification of AQP activity (e.g., phosphorylation) or changes in apoplastic flow (Maurel et al., 2008; Zhang et al., 2019). This complexity aligns with the well-established, context-dependent role of ABA in regulating plant water relations. While our functional data suggest a post-translational mode of action for ABA, future studies employing phosphoproteomics or PIP-specific antibodies are needed to directly confirm the phosphorylation status and abundance of PIP proteins in this context. Furthermore, the observed negative feedback loop, where exogenous ABA suppressed its own biosynthesis genes in the hydrated zone (**Figures 6A, D**), highlights the precision and autonomy of local ABA homeostatic control.

### An integrated signaling model for hydraulic compensation in APRI

Synthesizing these findings, we propose a refined model for the active hydraulic compensation in PRI (**Figure 8**). JA, synthesized in leaves, which is induced by the signals initiated by the drying of the dehydrated root zone is translocated to the hydrated roots as a systemic master signal. Here, it activates a dual pathway: (1) activation of *RBOHC*-mediated H_2_O_2_ production, which further contributes to up-regulate *GhPIP1;5* and *GhPIP2;8*, and thereby enhancing root *L*. (2) JA induces local ABA accumulation, which fine-tunes *L* through post-translational mechanisms. This coordinated, JA-centric network ensures a rapid and robust enhancement of water uptake in the hydrated root fraction.

This model extends the classical PRI theory beyond ABA-mediated stomatal closure (“reducing outflow”) by detailing the “enhancing inflow” mechanism. It offers novel targets for crop improvement. Breeding or engineering for enhanced JA sensitivity/biosynthesis in roots, or for alleles of key *PIP* genes like *GhPIP1;5* and *GhPIP2;8* that are more responsive to this signaling cascade, could amplify the water-saving benefits of PRI (Zhang et al., 2024). Furthermore, integrating APRI with nutrient management (e.g., potassium fertilization) could yield synergistic benefits for stress tolerance and yield under water scarcity (Elshamly and Abaza, 2024).

### Limitations and future perspectives

It is important to note that our analysis represents a focused snapshot at 24 hours after treatment initiation. While this time point captured key signaling and physiological responses, it does not elucidate the full temporal dynamics of the network. Future time-course studies are warranted to determine how the hierarchical relationships between JA, H_2_O_2_, and ABA evolve throughout a complete PRI irrigation cycle, distinguishing transient ‘emergency’ signals from sustained acclimation.

## Conclusion

In conclusion, our integrated physiological and molecular analysis demonstrates that the superior water use efficiency of PRI is underpinned by an active, JA-driven signaling network in the hydrated root zone. This network integrates H_2_O_2_ as a key downstream effector and ABA as a fine-tuning modulator to synergistically upregulate both the expression and activity of aquaporins, thereby significantly enhancing root *L*. This compensatory mechanism ensures effective water capture and hydration maintenance under partial root-zone drying. Thus, it elucidates the physiological basis for the consistently observed improvements in crop water productivity under field APRI regimes. These insights deepen our understanding of plant adaptation to heterogeneous water environments and provide a theoretical foundation for developing novel management strategies or genetic tools to further optimize water productivity in agriculture.

## Data Availability

The datasets generated and analysed during the current study are available in the Zenodo repository, https://doi.org/10.5281/zenodo.18856881.

## Supplementary material

Supplementary Figure 1 Schematic diagram of the experimental system simulating APRI, alternate partial root-zone drip irrigation. **(A)** Field configuration of APRI under mulched drip irrigation. **(B)** Stratified rhizobox system used in this study. The system consists of an upper compartment (20×20×5.5 cm) and a lower compartment (20×30×5.5 cm), allowing independent soil moisture management in each zone to mimic the APRI cycle.

Supplementary Figure 2 Validation of leaf-derived JA/JA-Ile translocation to roots under PRI. **(A-C)** Relative expression of JA biosynthesis genes, JA, and JA-Ile content in leaves at 24 HAT under different irrigation regimes. **(D, E)** JA and JA-Ile content in the hydrated roots of PRI plants following foliar application of JA or its biosynthesis inhibitor ETYA. FI, Full Irrigation; PRI, Partial Root-zone Irrigation. Data are presented as mean ± SD (n=6). Different lowercase letters indicate statistically significant differences (P < 0.05) among treatments within each panel as determined by Games-Howell *post-hoc* test.

Supplementary Table 1 Summary of Statistical Analyses for Data Presented in **Figures 1**-**7** and **Supplementary Figures**.
