# Supplementary material for: JA-mediated H2O2 and ABA signaling enhances root hydraulic conductance in cotton under partial root-zone irrigation
Source: Front Plant Sci. 2026 Apr 2;17:1784771. doi: 10.3389/fpls.2026.1784771 (PMC13082963; doi:10.3389/fpls.2026.1784771)
Supplement: Supplementary Figure 1 — Schematic diagram of the experimental system simulating APRI, alternate partial root-zone drip irrigation. (A) Field configuration of APRI under mulched drip irrigation. (B) Stratified rhizobox system used in this study. The system consists of an upper compartment (20×20×5.5 cm) and a lower compartment (20×30×5.5 cm), allowing independent soil moisture management in each zone to mimic the APRI cycle. [file DataSheet1.docx]

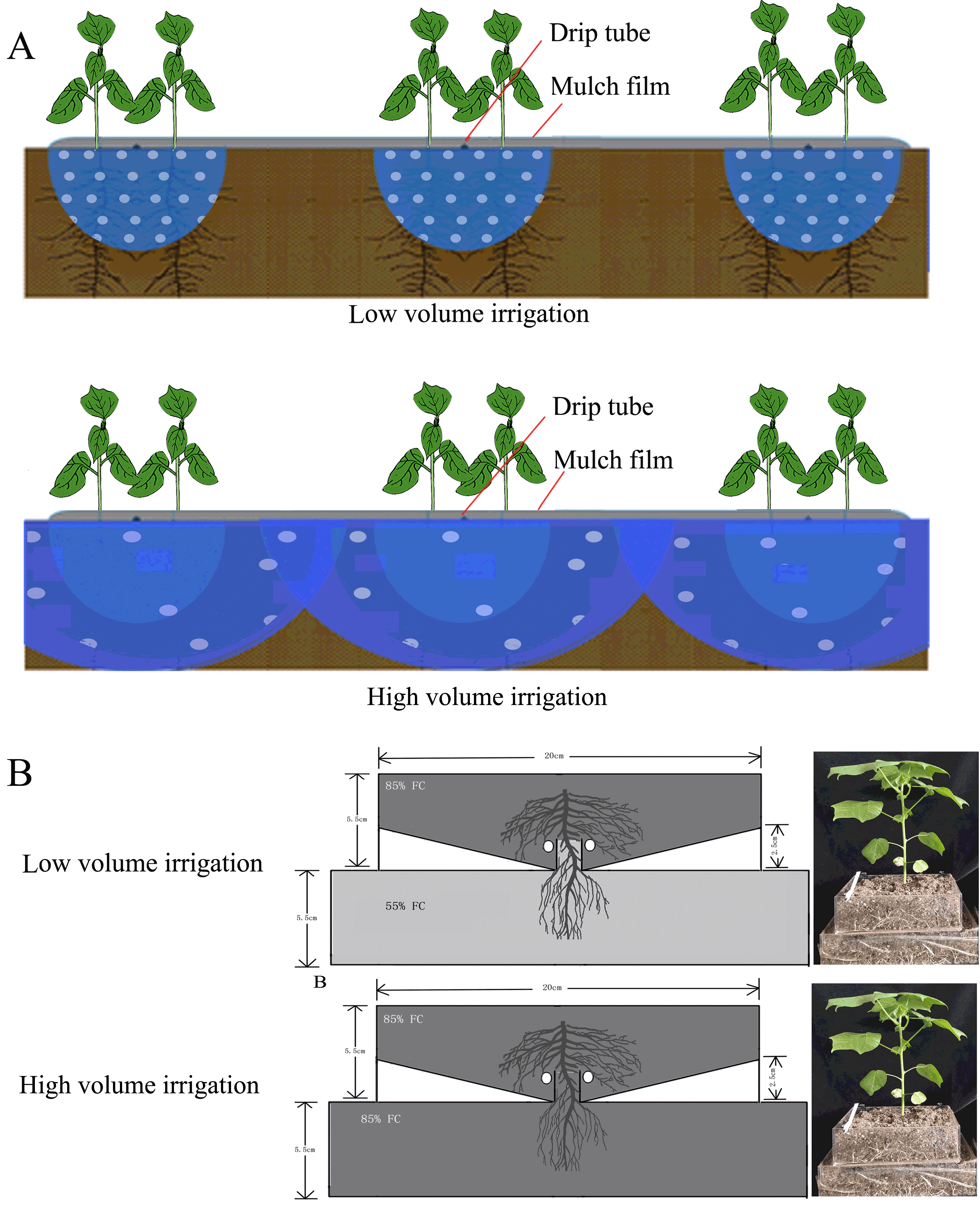


Supplemental Figure S1. Schematic diagram of the experimental system simulating alternate partial root-zone drip irrigation (APRI). (A) Field configuration of APRI under mulched drip irrigation. (B) Stratified rhizobox system used in this study. The system consists of an upper compartment (20×20×5.5 cm) and a lower compartment (20×30×5.5 cm), allowing independent soil moisture management in each zone to mimic the APRI cycle.

Supplemental Figure S2. Validation of leaf-derived JA/JA-Ile translocation to roots under PRI. (A-C) Relative expression of JA biosynthesis genes, JA, and JA-Ile content in leaves at 24 HAT under different irrigation regimes. (D, E) JA and JA-Ile content in the hydrated roots of PRI plants following foliar application of JA or its biosynthesis inhibitor ETYA. FI: Full Irrigation; PRI: Partial Root-zone Irrigation. Data are presented as mean ± SD (n = 6 independent rhizoboxes). Different lowercase letters indicate statistically significant differences (P < 0.05) among treatments within each panel.
